# Immunological Responses, Expression of Immune-Related Genes, and Disease Resistance of Rainbow Trout (*Oncorhynchus mykiss*) Fed Diets Supplied with Capsicum (*Capsicum annuum*) Oleoresin

**DOI:** 10.3390/ani14233402

**Published:** 2024-11-25

**Authors:** Sevdan Yilmaz, Osman Nezih Kenanoğlu, Sebahattin Ergün, Ekrem Şanver Çelik, Mert Gürkan, Elsayed Eldeeb Mehana, Hany M. R. Abdel-Latif

**Affiliations:** 1 Department of Aquaculture, Faculty of Marine Sciences and Technology, Çanakkale Onsekiz Mart University, Çanakkale 17100, Türkiye; sergun@comu.edu.tr; 2 Department of Food Engineering, Faculty of Engineering and Architecture, Kastamonu University, Kastamonu 37150, Türkiye; okenanoglu@kastamonu.edu.tr; 3 Department of Marine Biology, Faculty of Marine Sciences and Technology, Çanakkale Onsekiz Mart University, Çanakkale 17100, Türkiye; sanver_celik@comu.edu.tr; 4 Department of Biology, Faculty of Arts and Sciences, Çanakkale Onsekiz Mart University, Çanakkale 17100, Türkiye; mertgurkan@comu.edu.tr; 5 Department of Pathology, Faculty of Veterinary Medicine, Alexandria University, Alexandria 22758, Egypt; elsayedhamouda@gmail.com; 6 Department of Poultry and Fish Diseases, Faculty of Veterinary Medicine, Alexandria University, Alexandria 22758, Egypt

**Keywords:** Capsicum, hematology, challenge, immunity, gene expression

## Abstract

A 45-day feeding study was carried out to assess the immune-stimulatory effects of capsicum oleoresin when added to rainbow trout diets. A total of 450 fish (mean weight: 155.20 ± 1.96 g) were distributed into 400 L tanks (30 fish/tank) across five experimental groups: control (CT, 0%), C7 (0.7%), C14 (1.4%), C21 (2.1%), and C28 (2.8%). Each group consisted of three replicate tanks. At the end of this period, hemato-biochemical parameters, innate immune responses, and immune-related gene expression levels were evaluated, and a histological examination of head kidney and liver sections was conducted. Finally, fish in all groups were challenged with *Lactococcus garvieae* and observed for an additional 20 days. The results revealed that oleoresin supplementation enhanced the immune responses of the treated fish, which was evidenced by the increased globulin, total protein, respiratory burst activity, and total immunoglobulin levels. The highest expression levels of the *il-8*, *il-1β*, *TGF-β*, and *SAA* genes was noticed in the C7 group, as compared with the results for the other groups. The *IgT* gene expression levels were higher in all experimental groups than in the CT group, and this increase was at the highest level in the C28 group. Following the bacterial challenge, all experimental groups displayed higher survival rates compared to that of the CT group. These values were 75.93, 72.22, 46.30, 33.33, and 29.63% in the C7, C14, C21, C28, and CT groups, respectively, with the C7 group displaying the highest survival rate among the groups. The histological examination of liver and head kidney tissues revealed that higher doses (in the C21 and C28 groups) showed an increase in cytoplasmic vacuolization, which causes adverse effects on fish health. However, the C7 group displayed normal histological structure in both tissues. Taken together, the most favorable immune responses were achieved in the C7 group, suggesting that 0.7% oleoresin could be applied to rainbow trout to boost immunity and protect the fish from diseases.

## Simple Summary

Phytochemicals are plant-based bioactive compounds obtained (extracted) from various sources including nuts, fruits, herbs, whole grains, and vegetables. Research has proved that these compounds exhibit a vast number of benefits and advantages. Capsicum oleoresin is an oily organic resin that is extracted from the fruit of plants in the genus Capsicum. It is a plentiful source of phytochemicals that exhibit phenolics with potent antioxidant activities. This study was carried out to assess the immune-stimulatory effects of capsicum (*Capsicum annuum*) oleoresin when added to rainbow trout diets.

## 1. Introduction

Antibiotic use in aquaculture is prohibited in many countries throughout the globe [1]. This is because of the possibility of bacterial resistance against antibiotics, with potential consumer concerns [2]. The persistence of drug residues in fish meat, environmental pollution, and high costs are among the other reasons for this regulation [3]. To eliminate these problems, preventive measures and safe alternatives must be considered [4] to control bacterial diseases [5]. Among the preventive measures, the trend of using immunostimulants has attracted significant attention from many researchers in recent years [6]. Immunostimulation, as the name suggests, refers to the activation of the immune responses of the fish before they become diseased [7].

The use of medicinal plants as immunostimulants contributes to the development of specific and non-specific immunity mechanisms, as well as increases resistance to fish diseases [8,9,10]. It is evident that the active components of medicinal plants play certain roles in the activation of various components of the immune system, such as lysozyme, complement, B and T lymphocytes, natural killer cells, and phagocytosis; thus, the plants and their byproducts could strengthen the fish immune system [11,12]. Medicinal plants are effective antibiotic alternatives because they are harmless, since they are natural, organic products. Moreover, they are also eco-friendly, cheap, and easily processable [13].

Since ancient times, hot pepper has been utilized as an additive to preserve food and attract consumers with its color. Although it is typically used in powder form (paprika), it has recently been used in the form of pepper oil and oleoresin [14]. *Capsicum annuum* is recognized throughout the world as one of the most widely utilized spices owing to its distinctive and robust flavor. The oleoresin of *C. annuum* (CAO) is a natural extract, rich in carotenoids, derived from various *C. annuum* cultivars [15]. The primary bioactive compounds in CAO are capsaicinoids, with capsaicin identified as the major component [16]. Additionally, CAO serves as an excellent source of essential vitamins, particularly ascorbic acid and vitamin E, and contains pigments such as xanthophylls. Capsaicinoids also exhibit significant antioxidant and antimicrobial properties [17]. Due to its content of β-carotene and other carotenoids, hot pepper may be a useful in scavenging free radicals [18]. Research studies have shown that hot pepper is among the plants that exhibit potent antioxidant potential [19,20]. Our recently published study uncovered the potential role of a diet supplemented with capsicum oleoresin (7–14 g/kg) to enhance the growth, intestinal histomorphometry, and sensory characteristics of rainbow trout [21]. To our knowledge, no previous studies have investigated the dietary effects of capsicum oleoresin on the immune responses and disease resistance of fish. Therefore, this study aimed to evaluate its effects on the hemato-biochemical indices, innate immune parameters, head kidney immune-related gene expression responses, and bacterial infection resistance of rainbow trout fed with capsicum oleoresin-supplemented feeds. The results of this study could help to enhance the functionality of aquafeed.

## 2. Materials and Methods

### 2.1. Fish and Experimental Setup

This study was carried out at Çanakkale Onsekiz Mart University, Faculty of Marine Sciences and Technology, Living Resources Production Unit. A total of 450 rainbow trout, weighing 155.20 ± 1.96 g (mean ± SD), were used in the experimental trial. Prior to the feeding trial, fish were allowed to be acclimated to the laboratory environment for 15 days. The experiment was conducted in a closed-circuit system. The setup consisted of 15 plastic tanks with a volume of 400 L each. These tanks were connected to a settling pool, coarse filtration, sand filter, biological filter, and a heating–cooling unit (Tuna Mac^®^, Çanakkale, Türkiye). Water was replaced daily at the rate of 10–15%. The photoperiod in the trial unit was maintained at 12 h of light/12 h of dark with the help of an automatic timer. A total of 30 fish were placed into each tank, with three tanks per group. The control group (CT) received a basal feed, without additives, as described in our study [22]. The experimental groups received the feeds containing different levels of capsicum oleoresin (0.7, 1.4, 2.1, and 2.8%), according to our recently published study [21]; thus, the experimental groups were defined as C7, C14, C21, and C28, respectively.

### 2.2. Experimental Feeds

Capsicum oleoresin was purchased from a commercial company (Smart Kimya Tic. ve Danışmanlık Ltd. Şti. (İzmir, Türkiye). Isocaloric and isonitrogenous feeds were prepared (Table A1) to contain 43% protein and 16% crude lipid, in accordance with the rainbow trout’s nutritional requirements [22]. Experimental feeds were prepared in a feed machine (La Monferrina—P3). First, the raw materials and the additives were mixed, forming a homogeneous mixture with the help of the mixing chamber of the feed machine. Second, water was added to the mixture, and the mixing process was continued until the feed reached the appropriate consistency. The feed was prepared using the 4 mm grinding plate. Afterwards, the drying process of the pellet feeds continued in a drying cabinet with air circulation at 40 °C until the moisture content reached 10%. Nutritional value analyses of the feeds and raw materials were performed according to AOAC procedures [23]. The feeding experiment was continued for 45 days.

### 2.3. Physical and Chemical Water Quality Analyses

The pH, temperature, dissolved oxygen (DO), and conductivity of water in experimental tanks were examined every other day throughout the study using an AZ 86031 combo water analyzer (AZ Instrument Corp., Taiwan, China). The total ammonia, nitrite, and nitrate levels were measured weekly by spectrophotometry (Optizen POP UV/VIS), with the help of commercial measurement kits (Spectroquant^®^ Merck Millipore, Darmstadt, Germany). Throughout the whole experimental period, the mean values of the variables were as follows: water temperature (15.9–16.5 °C), DO (7.3–7.9 mg L^−1^), conductivity (420–515 μs cm^−1^), pH (7.2–7.8), total ammonia (0.012–0.014 mg L^−1^), nitrite (0.02–0.03 mg L^−1^), and nitrate (0.16–0.22 mg L^−1^). These values were within the desirable limits for rainbow trout culture.

### 2.4. Blood and Tissue Sampling

Blood was collected from nine randomly selected fish per group (three fish per tank). The fish were anesthetized using clove oil at a concentration of 20 mg/L for 2 min to minimize handling stress [24]. Before sampling, the fins of the fish were wiped with cotton impregnated with 70% ethyl alcohol to clean the surface and to ensure that it was free of mucus secretions and did not mix with the blood. Blood collection from caudal veins was performed with 2.5 mL plastic syringes. The blood samples taken were placed in K2EDTA-containing tubes (MiniCollect^®^ K2E K2EDTA tubes, Greiner Bio-One, Kremsmünster, Austria), for hematological analyses and respiratory burst activity, and in MiniCollect^®^ Serum Separator Tubes (MiniCollect^®^ Greiner Bio-One, Kremsmünster, Austria), for serum biochemistry and total immunoglobulin analyses. To obtain serum, the samples collected in the relevant tubes were centrifuged at 5000× *g* for 5 min at room temperature. All obtained sera were stored at −80 °C for subsequent analysis. To sample the head kidney tissue for gene expression analyses, the fish whose blood collection was completed were euthanized by applying an overdose of clove oil (200 mg/L) [25]. Afterwards, the head kidney tissues taken from the carefully dissected fish were placed in PCR tubes, and RNA was later added.

### 2.5. Hematological Analyses

Red blood cell count (RBC), hematocrit (Hct), and hemoglobin (Hb) analyses were performed using an automatic blood counting (Mindray/BC 3000 Plus, Mindray Bio-Medical Electronics Co. Ltd., Shenzen, China), as previously conducted in *O. mykiss* [25].

### 2.6. Blood Biochemistry

Blood serum parameters, such as glucose, total protein, albumin, globulin, triglycerides, cholesterol, alkaline phosphatase (ALP), alanine transaminase (ALT), and aspartate transaminase (AST), which are health status indicators for fish, were evaluated via spectrophotometric readings using biochemical analysis kits (Bioanalytic Diagnostic Industry Co., Neuss, Germany).

### 2.7. Respiratory Burst Activity

The respiratory burst activity (RBA) of phagocytic cells was determined by spectrophotometric measurement of the response of NBT-positive cells to the 96-well adhesion of activated leukocytes in the blood, following the method of Stasiack and Bauman [26]. In brief, 50 µL of blood was placed in PLL-coated 96-well plates (Thermo Scientific, Nunc, #167008, Waltham, MA, USA; PLL solution: Sigma-Aldrich, #P4832, St. Louis, MO, USA) and then incubated at 25 °C for 1 h. After removing the supernatant, the wells were washed three times with HBSS (Sigma-Aldrich, #H6648, St. Louis, MO, USA). Then, 100 μL of 0.2% NBT in HBSS (Sigma-Aldrich, #N5514, St. Louis, MO, USA) was added and incubated for 1 h. Cells were then fixed with 100% methanol for 5 min, washed with 70% methanol, air dried, and treated with 60 μL of 2 M KOH (Sigma-Aldrich, #P5958, St. Louis, MO, USA) and 70 μL DMSO (Sigma-Aldrich, #D2650, St. Louis, MO, USA). The absorbance was measured at 620 nm using a plate reader (Thermo Multiskan Go, Waltham, MA, USA).

### 2.8. Lysozyme Activity

The lysozyme activity was determined using the method of Ref. [27]. A total of 25 μL of serum sample was added to 175 μL of *Micrococcus luteus* suspension (pH 5.8). The samples in 96-well plates were incubated at room temperature for 30 min. Readings were performed on a Multiskan microplate reader at 450 nm, and the lysozyme activity was calculated from the standard curve as µg/mL using a standard (L6876 Sigma, Lysozyme from chicken egg white, St. Louis, MO, USA).

### 2.9. Myeloperoxidase Activity

Myeloperoxidase activity (MPO) was determined by modifying the methods available in the literature [28]. A total of 10 µL of serum sample was diluted with 90 µL of HBSS solution. A solution containing 3,3′,5,5′-tetramethylbenzidine dihydrochloride and hydrogen peroxide was added to this mixture, and the reaction was terminated after 2 min by adding 35 µL of sulfuric acid. Then, readings were made on a Multiskan microplate reader at 450 nm [28].

### 2.10. Gene Expression

In the study, the head kidney tissues of the sampled fish were obtained at the end of the 45th day of the feeding experiment. Total RNA was isolated from these tissues, and then cDNAs were synthesized and measured. Afterward, the gene expression levels of the fish were detected using appropriate primers. Total RNAs from tissue samples were isolated using a Thermo Scientific GeneJET RNA Purification Kit (Waltham, MA, USA) according to the manufacturer’s instructions. Briefly, approximately 30 mg of kidney sample was taken for RNA isolation. The samples were isolated in 300 µL lysis buffer supplemented with β-mercaptoethanol using a homogenizer. Total RNA was obtained from the isolated samples by performing centrifugation steps, according to the manufacturer’s instructions. The purity and quantity of the obtained RNA were determined using Multiskan GO (Thermo Scientific, Waltham, MA, USA). The samples were then diluted to 15 ng/µL and used in cDNA synthesis. The RNA samples were stored at −80 °C until use. The cDNA was synthesized from the isolated total RNAs using the OneScript^®^ Plus cDNA Synthesis Kit (Applied Biological Materials Inc. (abm), Richmond, BC, Canada). In this process, the reaction mixture was formed by adding 1 µg of diluted RNA, 1 µL of oligo dT primer (10µM), 4 µL of 5x RT Buffer, and 1 µL of OneScript^®^ Plus RTase (Applied Biological Materials Inc. (abm), Richmond, BC, Canada), and the mixture is completed to 20 µL with nuclease-free water. cDNA synthesis was performed by incubating the sample for 15 min at 50–55 °C in a thermal cycler (Thermo Fischer Scientific, Waltham, MA, USA). The purity and quantity of the obtained cDNA were determined using Multiskan GO (Thermo Scientific, Waltham, Massachusetts, USA). Quantified samples were diluted to 20 ng/µL and stored at −20 °C until used for the determination of the gene expression levels. The qRT-PCR analysis was performed via the StepOnePlus TM Real-Time PCR system (Applied Biosystems, Waltham, MA, USA) using the 5x HOT FIREPol EvaGreen HRM Mix (Solis Bio-Dyne Co., Tartu, Estonia). Gene-specific primers were employed (Table A2). The expression of genes was quantified relative to β-actin as a reference gene using the 2-^ΔΔCt^ method [29].

### 2.11. Histological Analysis

In this study, head kidney and liver tissues of the fish samples (*n* = 9) were examined histologically. These tissues were removed from the fish after dissection at the end of the study and washed in physiological water. The tissues were divided into small pieces with a scalpel, based on their size. All tissue samples were fixed in Bouin’s solution for 12 h. The fixed tissues were stored in 70% ethyl alcohol at room temperature in the dark until the day of preparation. During the preparation process, tissue samples were passed through 80%, 85%, 90%, and 96% absolute alcohol; xylene; and a 1:1 xylene–paraffin series, respectively, and were embedded in paraffin. Paraffin blocks were cut to a 5 µm thickness on a Leica brand rotary microtome. For each fish sample, three different slides were prepared from the respective tissues. The slides were stained with Hematoxylin & Eosin for histological and histomorphological examinations. All slides were examined under an Olympus CX21 light microscope. In addition, histological photographs were captured using the Olympus Analysis LS program on a camera integrated with the microscope. For this analysis, five existing melanomacrophage (MMA) areas (µm^2^), in three randomly selected different parts on three slides prepared from the head kidney sections of the samples in all groups, were measured using the Olympus Analysis LS program. Ten hepatocytes were randomly selected from the liver section of each sample and were evaluated. In the measurements, three different slides were used for each of the control and application groups. The Olympus Analysis LS program was used for the hepatocyte measurements.

### 2.12. Bacterial Challenge Test

After the 45th day of the study, the remaining fish were used in a challenge test to evaluate the disease resistance of the capsicum oleoresin-supplemented fish. The fish began accepting the experimental diets 7–8 days after the challenge. The experimental groups were fed the same diets they received prior to the challenge, continuing with these diets until the end of the challenge period. In the challenge test, *Lactococcus garvieae* SY-LG1 pathogen (NCBI GenBank Accession No. KY118086.1) was used. The pathogen was previously isolated from diseased rainbow trout. *L. garvieae* was incubated in BHB medium for 16 h at 24 °C. The bacterial cells were washed with PBS, and the cell density was adjusted to (1.2 × 10^7^ CFU/mL in PBS) (previously calculated LD50 dose) [22]. A total of 18 fish from each tank (54 fish per group) were intraperitoneally injected, using an insulin syringe, with 100 µL of PBS solution containing the pathogen. Mortality rates were recorded for the following 20 days. *L. garvieae* was re-isolated from the dead fish and was identified using conventional microbiological tests, as well as 16S rDNA analysis.

### 2.13. Statistical Analyses

One-way ANOVA was applied to the data obtained from the analyses. Tukey’s multiple comparison test was used when the data were normally distributed and homogeneous. The Kruskal–Wallis test was used to compare non-normally distributed and homogeneous data, and the Tamhane test was employed to compare non-homogeneous data. Statistical analyses were performed at the *p* < 0.05 significance level using the SPSS 19 (IBMM SPSS Statistics 19) program.

## 3. Results

### 3.1. Hematological Parameters

The changes observed in RBCs count, Hb content, and Hct levels of the control (CT) and experimental groups at the end of the feeding trial are given in Table 1. According to the results, while there were no significant differences in RBCs count and Hct values between all groups (*p* > 0.05), the Hb values in the C14 group decreased significantly compared to those of the CT group (*p* < 0.05).

### 3.2. Serum Biochemical Parameters

**Figure 1:**
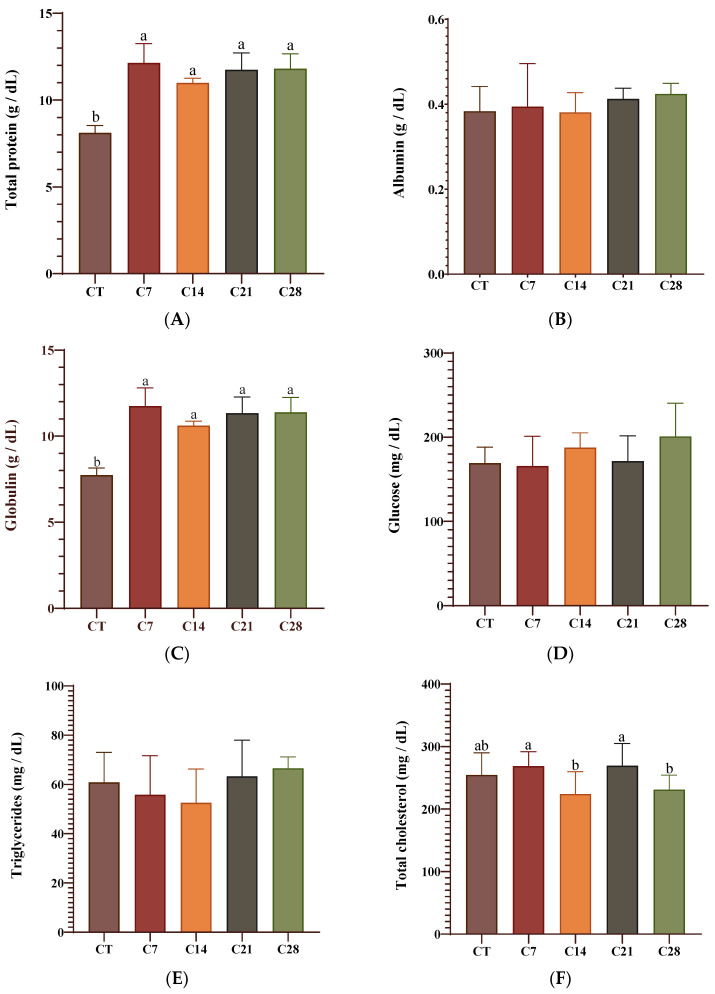
A panel of the serum biochemical parameters including total protein (**A**), albumin (**B**), globulin (**C**), glucose (**D**), triglycerides (**E**), and total cholesterol (**F**) of rainbow trout fed diets supplied with different levels of capsicum oleoresin for 45 days. Values are presented as means ± S.D. (*n* = 9). The bars assigned with different letters denote statistically significant differences between the control (CT) and treated groups (*p* < 0.05).

**Figure 2:**
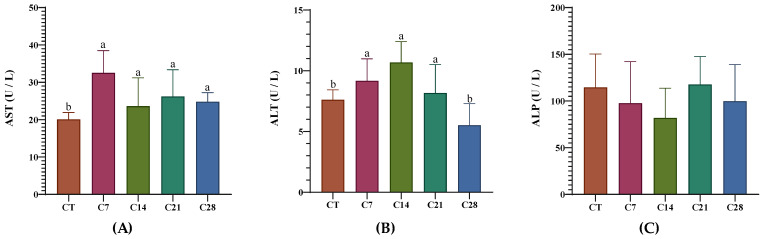
A panel of liver function enzymes including AST (**A**), ALT (**B**), and ALP (**C**) enzymes of rainbow trout fed diets supplied with different levels of capsicum oleoresin for 45 days. Values are presented as means ± S.D. (*n* = 9). The bars assigned with different letters denote statistically significant differences between the control (CT) and treated groups (*p* < 0.05).

The serum biochemical parameters of the rainbow trout fed the control and experimental diets are presented in Figure 1 and Figure 2. It was found that the albumin, glucose, triglycerides, and ALP levels did not differ significantly between the experimental groups. On the other hand, in all experimental groups, the total protein and globulin values increased compared to those of the control group (*p* < 0.05). Moreover, the cholesterol values in groups C14 and C28 significantly decreased compared to those of the other groups, including the control (*p* < 0.05). Of interest, the ALT and AST values increased significantly in all experimental groups compared to those of the CT group (*p* < 0.05; Figure 2).

### 3.3. Immune Responses

**Figure 3:**
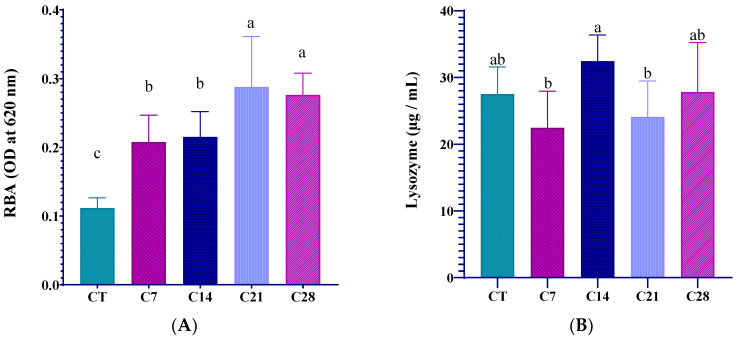

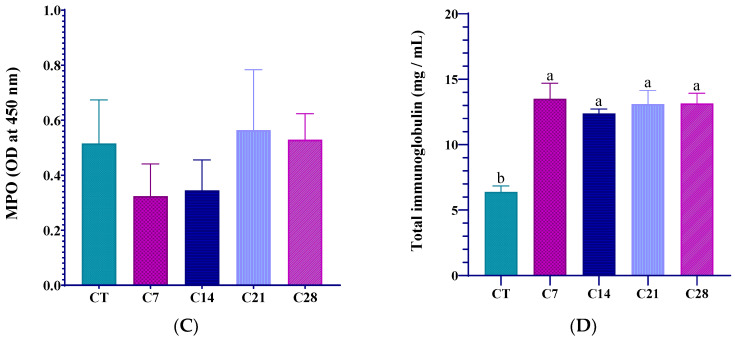
A panel of serum immune indices including RBA (**A**), lysozyme (**B**), MPO (**C**), and total immunoglobulin (**D**) of rainbow trout fed diets supplied with different levels of capsicum oleoresin for 45 days. Values are presented as means ± S.D. (*n* = 9). The bars assigned with different letters denote statistically significant differences between the control (CT) and treated groups (*p* < 0.05).

The serum immune indices of rainbow trout fed diets supplied with different levels of capsicum oleoresin for 45 days are given in Figure 3. According to the results, it was found that respiratory burst activity and total immunoglobulin values increased significantly in all experimental groups compared to those of the CT group (*p* < 0.05). On the other hand, there was no significant increase in myeloperoxidase enzyme activities compared to those of the CT group (*p* > 0.05). Serum lysozyme activity was increased significantly in the C14 group compared to that of the other groups.

### 3.4. Expression Levels of Immune-Related Genes

**Figure 4:**
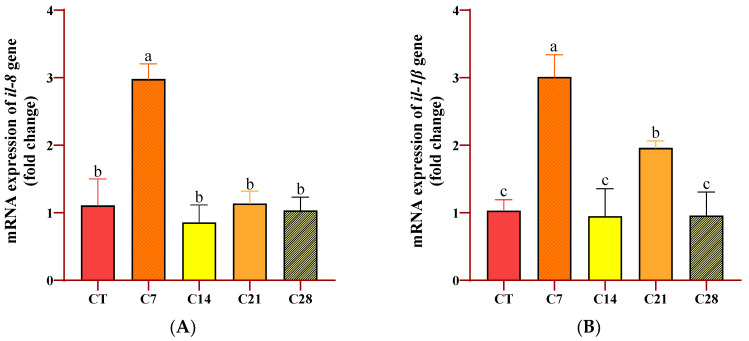

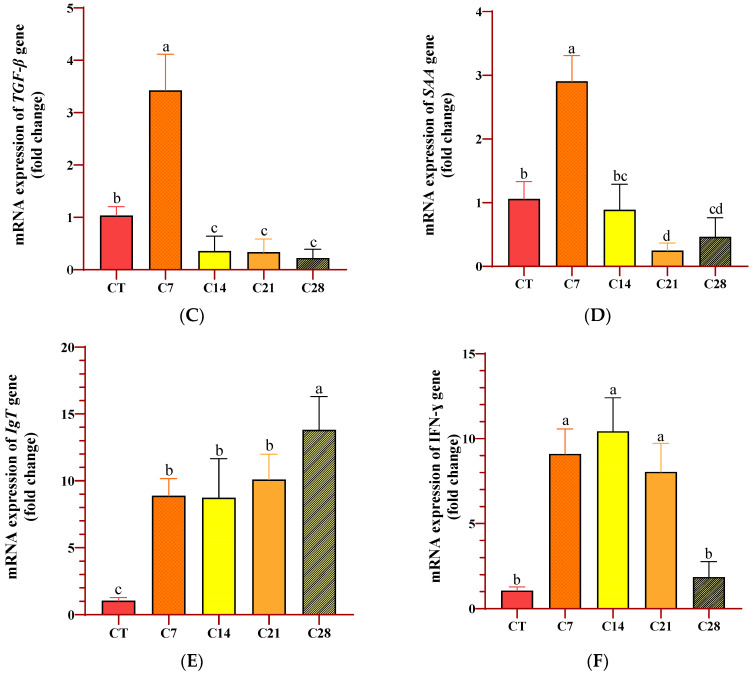
Expression responses of immune-related genes including il-8 (**A**), *il-1β* (**B**), *TGF-β* (**C**), *SAA* (**D**), *IgT* (**E**), and *IFN-ɣ* (**F**) genes of rainbow trout fed diets supplied with different levels of capsicum oleoresin for 45 days. Values are presented as means ± S.D. (*n* = 9). The bars assigned with different letters denote statistically significant differences between the control (CT) and treated groups (*p* < 0.05).

The expression levels of immune-related genes in the head kidney of rainbow trout after the feeding duration are presented in Figure 4. The results indicate that the highest increase in *Il-8*, *Il-1β*, *TGF-β*, and *SAA* gene expression levels was noticed in the C7 group compared to the levels of all other groups (*p* < 0.05). Moreover, *IgT* gene expression levels were higher in all experimental groups than in the CT group, and this increase was at the highest level in the C28 group (*p* < 0.05). However, regarding the *IFN-ɣ* gene, while the expression level of the C28 group was at a similar level to that in the CT group, with no significant differences (*p* > 0.05), there was a significant increase in the other experimental groups compared to the levels in the CT and C28 groups (*p* < 0.05).

### 3.5. Histological Findings

#### 3.5.1. Head Kidney Sections

**Figure 5:**
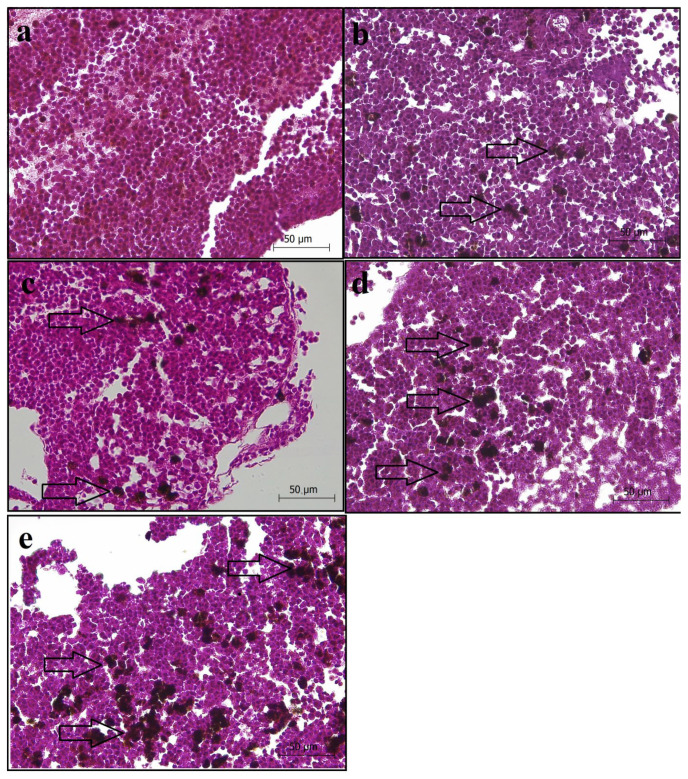
Photomicrographs of the head kidney sections of rainbow trout fed diets supplied with different levels of capsicum oleoresin for 45 days. (**a**) Control (CT), (**b**) C7, (**c**) C14, (**d**) C21, and (**e**) C28. Arrows show melanomacrophage aggregation. ×40 magnification; H&E.

In the head kidney sections, no histological abnormality was detected in the sections taken from the CT group, whereas the blood supply within the sinusoids, the melanomacrophage density, and the cell borders were normal (Figure 5a). In the C7 group (Figure 5b), and in other experimental groups including C14, C21, and C28 (Figure 5c–e), there was an increase in the melanomacrophage density, which is thought to be due to capsicum oleoresin supplementation. The data showed that melanomacrophage density was more evident, especially in the C21 and C28 groups. In addition, a decrease in the density of the connective tissue elements was detected in the head kidney sections of fish belonging to the C21 and C28 groups.

#### 3.5.2. Liver Sections

**Figure 6:**
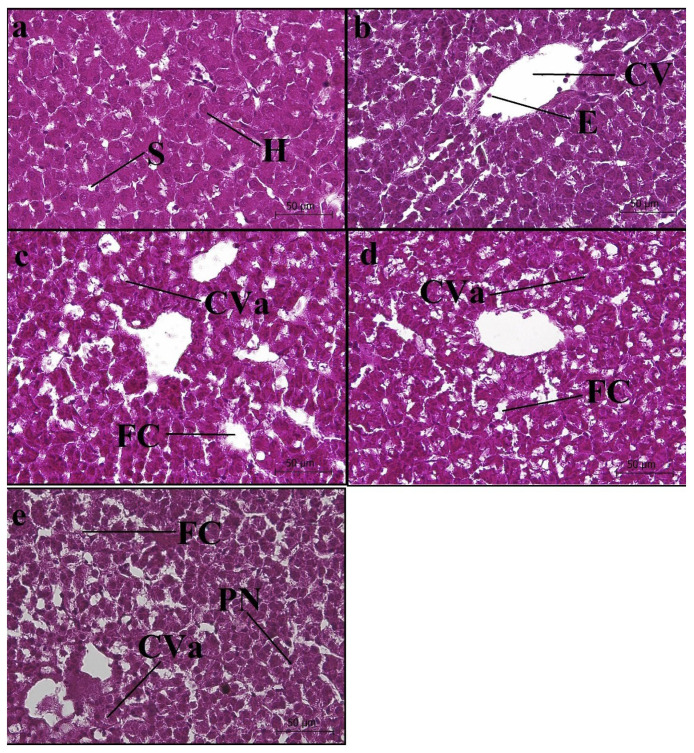
Photomicrographs of the liver sections of rainbow trout fed diets supplied with different levels of capsicum oleoresin for 45 days. (**a**) Control (CT), (**b**) C7, (**c**) C14, (**d**) C21, and (**e**) C28 (S: sinusoid; H: hepatocyte; E: erythrocyte; CV: central vena; FC: fatty change; CVa: cytoplasmic vacuolization; PN: pyknotic nucleus). ×40 magnification; H & E.

In the CT group, no histological changes were detected in the liver sections, and no anomalies were observed in the diameters and endothelium of the central vein, portal vein, hepatic artery, and capillary. Moreover, the hepatocytes displayed a normal appearance and size. Furthermore, no hypertrophic and/or hyperplasic areas were observed in the sections. The sinusoidal cavities were of normal width (Figure 6a). An increase in the sinusoidal cavity was detected in the liver sections of the C7 group (Figure 6b). Similarly, increases in the sinusoidal cavity were observed in the C14, C21, and C28 groups. Cytoplasmic vacuolization was found to be prevalent in the hepatocytes of the C14 group (Figure 6c) and were clearly widespread in the sections of the C21 and C28 groups; diffuse fatty changes were observed in these sections (Figure 6d,e).

### 3.6. Histomorpholometric Results

The histomorphometric changes and their observed frequency in the liver and head kidney sections of the fish in the CT and treatment groups are presented in Table 2. Significant differences were detected between the CT and capsicum-supplemented groups in terms of mean melanomacrophage aggregation area (*p* < 0.05). The melanomacrophage aggregation increased, especially in the C21 and C28 groups. However, no differences were detected in the mean hepatocyte diameter between the CT and capsicum-supplemented groups (*p* > 0.05).

### 3.7. Results of the Bacterial Challenge Test

The MR, SR, and RPS of fish obtained after the bacterial challenge test are presented in Table 3. The results show that the highest SR was observed in the C7 group, followed by the C14, C21, C28, and CT groups, respectively. Their values were 75.93, 72.22, 46.30, 33.33, and 29.63% in the C7, C14, C21, C28, and CT groups, respectively.

## 4. Discussion

Hematological parameters are excellent bio-indicators for assessing the health status and nutritional condition of fish [30]. Moreover, changes in RBCs count, Hct, Hb, or erythrocyte indexes provide information regarding the health status of fish [31]. According to the present study’s results, while there was no difference in the RBCs count and Hct values between all groups, it was determined that the Hb values in the C14 group were lower than those in the CT group. Contrary to our research findings, it was reported that a 1% supplementation of hot pepper extract, which they tested on rainbow trout fry, did not cause any decrease in the mentioned hematological parameters; in fact, lower concentrations of the extract (0.25% and 0.5%) significantly increased RBC and Hb values [32]. It is evident that the hematological parameters vary depending on the different red pepper byproducts used in the diet. However, in our study, the fact that Hb values decreased only in the C14 group and not in any other experimental group suggests that the difference in the Hb value was not caused by the hot pepper oleoresin. It could be attributed to the individual differences of the fish.

In the present study, the total protein and globulin increased significantly in all of the capsicum oleoresin-supplemented groups compared to the levels in the CT group. Moreover, a significant decrease was observed in serum cholesterol levels in the C14 and C28 groups. Firouzbakhsh et al. [32] reported that a significant increase was determined in the total protein levels of the fish fed with hot pepper extract. However, these researchers demonstrated that albumin levels also increased in the experimental groups, and there was no significant difference in globulin levels. Moreover, Parrino et al. [33] found that a 1‰ concentration of red pepper oil, which they tested as a feed additive in rainbow trout, reduced glucose, cholesterol, and ALP values compared to those of the CT group, while 2‰ and 4‰ concentrations only decreased the glucose values. These researchers also reported that a 6‰ concentration decreased the total protein level and increased glucose, albumin, and LDH values compared to those of the CT group. These results show how different derivatives of the same product (oil, oleoresin, extract, etc.) affect the health status of the fish differently.

The respiratory burst activity of phagocytes is an indicator of an organism’s ability to neutralize reactive oxygen species (ROS) [34]. Immunoglobulins are antibodies that are used by the organism in processes of identifying and eliminating foreign antigens [35]. Likewise, lysozyme is an important molecule in the innate defensive mechanisms of fish, and it plays an active role in the elimination of pathogens [36,37]. The myeloperoxidase (MPO) enzyme plays an important role in protecting against the bacterial infections of fish, as macrophages can receive MPO from neutrophils, and this may enhance their bactericidal activity [38]. In this study, although the lysozyme and MPO activities were similar in all groups, there were significant increases in the respiratory burst activity and total immunoglobulins values of the capsicum oleoresin-supplemented groups compared to those of the CT group, indicating that the immune responses of the fish were positively affected. Similarly, it was demonstrated that serum lysozyme activity in rainbow trout increased dose-dependently with 0.25%, 0.5%, and 1% red pepper extract supplementation at the end of a 30-day feeding study [32].

The gene expression levels of cytokines and other immune-related molecules are investigated in immunostimulant studies, as they are reliable tools determined using state-of-the-art techniques. Serum amyloid A (SAA) functions as an opsonin, enhances phagocytosis, and plays a role in the mediation of the inflammatory response [39,40]. Interleukin-8 (IL-8) is a cytokine that is present in many teleost fish, and it plays a significant role in bacterial infection by inducing inflammation [41]. Interleukin-1 beta (IL-1β) is mainly produced by macrophages and monocytes and acts as a proinflammatory cytokine that play an essential role in protecting against microbial infections. It also helps in the secretion of various other cytokines [42]. Moreover, transforming growth factor beta (TGF-β) plays roles in the regulation of lymphocyte differentiation and proliferation. It also plays a role in the initiation and continuation of the inflammatory response [43]. Interferon gamma (IFN-γ) is another critical cytokine of the immune system. It is specifically produced by the immune cells and plays a key role in the cell-mediated immune responses [44]. Tumor necrosis factor alpha (TNF-α) is involved in the regulation of immune cells and systemic inflammation. It plays a vital role in the pro- and anti-inflammatory cytokines’ network by inducing the production of itself and several other critical cytokines [45]. Immunoglobulin M (IgM) is the main antibody of most teleost fishes [46] and primarily operates in the infection response [47]. Another immunoglobulin, immunoglobulin T (IgT), which was recently discovered from fish by Ref. [48], mainly functions as the first-line defense against pathogens because it is present in the surfaces of fish [49]. In the present study, it was observed that the *IL-8*, *IL-1β*, *TGF-β*, and *SAA* gene expression levels were significantly higher in the group supplemented with 0.7% oleoresin compared to the other groups at the end of the experiment. Moreover, *IgT* and *IFN-γ* expression levels were significantly increased in all the capsicum oleoresin-supplemented groups. As in our results, Ibrahim et al. [50] reported that 0.4%, 0.8%, and 1.6% supplementation of hot pepper extract significantly increased the *TGF-β* gene expression levels in Nile tilapia compared to those of the CT group. On the contrary, these authors also stated in the same research that the *IL-1β* and *IL-8* gene expression levels significantly decreased in all experimental groups. In another study, Khieokhajonkhet et al. [51] determined that red pepper extracts increased the gene expression levels of *IGF-1*, *IGF-2*, *TNF-α*, and *IL-10* in goldfish (*Carassius auratus*). 

Histology is an important tool to assess the fish health status [52,53]. In the present study, it was observed that the capsicum supplementation at the rates of 2.1 and 2.8% adversely affected fish head kidney and liver sections. However, it can be inferred that the histological findings in the 0.7% capsicum oleoresin group were normal. This suggests that higher doses of capsicum oleoresin could be harmful to rainbow trout. These results are in concordance with our previously published study, wherein higher dietary doses of dietary capsicum oleoresin induced vacuolations and intestinal lesions in treated rainbow trout [21].

The challenge test is a viable tool for assessing the immune responses and disease resistance abilities of the treated fish [54]. In the present study, the results showed that the highest survival rate was recorded in the C7 group, followed by the C14, C21, C28, and CT groups, respectively, indicating that lower doses of capsicum oleoresin provided more immune strength and more resistance against bacterial challenge. These results are in agreement with the results showing increased innate immune responses and immune gene expression levels in the C7 group. Similarly, Diler et al. [55] found that oregano essential oil supplementation significantly increased the survival rate of rainbow trout after the challenge test with *L. garvieae*. Similarly, Firouzbakhsh et al. [32] reported that the rainbow trout fed with red pepper extract displayed significantly lower mortality rates against *Yersinia ruckeri* in comparison with the rates observed in the CT group. Latterly, it was demonstrated that feeding rainbow trout with a phytogenic diet significantly enhanced their survival after challenge with a pathogenic *L. garvieae* [22].

## 5. Conclusions

In summary, it can be suggested that 0.7% capsicum oleoresin supplementation for 45 days significantly stimulated the innate immune responses and disease resistance of rainbow trout, with no side effects on the fish tissues, suggesting a possible application in aquafeed for enhancing the sustainability of aquaculture and ensuring a disease-free and antibiotic-free environment. Future studies are needed to investigate the effects of dietary capsicum oleoresin supplementation to determine dosage strategies and different supplementation periods and sampling intervals for improving fish health.

## Figures and Tables

**Table 1:** Hematological parameters of rainbow trout fed diets supplied with different levels of capsicum oleoresin for 45 days.

Parameters	Experimental Groups				
	CT (Control)	C7	C14	C21	C28
Red blood cells (RBCs; ×10^6^ per mm^3^)	2.46 ± 0.11 ^a^	2.31 ± 0.06 ^a^	2.32 ± 0.08 ^a^	2.30 ± 0.09 ^a^	2.37 ± 0.08 ^a^
Hemoglobin (Hb; g/dL)	9.49 ± 0.68 ^a^	7.96 ± 0.33 ^ab^	7.87 ± 0.34 ^b^	8.01 ± 0.32 ^ab^	7.98 ± 0.54 ^ab^
Hematocrit (Hct; %)	34.51 ± 1.39 ^a^	31.96 ± 0.81 ^a^	33.01 ± 1.00 ^a^	31.91 ± 0.94 ^a^	33.16 ± 1.23 ^a^

Data are presented as means ± SD (*n* = 9). Values with different superscript letters in the same row are considered as statistically different within the groups (*p* < 0.05).

**Table 2:** Histological changes and their observed frequency in rainbow trout fed diets supplied with different levels of capsicum oleoresin for 45 days (+: rare; ++: moderate; +++: frequent).

Tissue	Histological Changes and Measurements	Experimental Groups				
		CT (Control)	C7	C14	C21	C28
**Liver**	Cytoplasmic vacuolization	**-**	**+**	**++**	**+++**	**+++**
	Mononuclear cell infiltration	**-**	**+**	**+**	**+**	**+**
	Pyknotic nucleus	**-**	**+**	**+**	**++**	**+++**
	Sinusoidal cavity	**-**	**+**	**+**	**++**	**+++**
	Fatty changes	**-**	**+**	**+**	**++**	**+++**
	Mean hepatocyte diameters (µm)	8.66 ± 0.41	8.48 ± 0.23	8.42 ± 0.19	8.74 ± 0.33	8.69 ± 0.38
**Head Kidney**	Melanomacrophage aggregation (MMA)	**+**	**++**	**++**	**+++**	**+++**
	Mean MMA area values	6.21 ± 1.18 ^a^	12.15 ± 1.47 ^b^	11.87 ± 1.17 ^b^	22.74 ± 3.12 ^c^	26.14 ± 2.86 ^c^

Statistical differences between means are indicated by superscript letters. Measurements are given as mean ± SD. -; negative, +; positive.

**Table 3:** Mortality and relative percent survival rates of rainbow trout fed diets supplied with different levels of capsicum oleoresin for 45 days and then challenged with *Lactococcus garvieae* and observed for 20 days.

	Experimental Groups				
	Control (CT)	C7	C14	C21	C28
Total number of fish challenged	54	54	54	54	54
Number of dead fish	38	13	15	29	36
Mortality rate (MR; %)	70.37	24.07	27.78	53.70	66.67
Survival rate (SR; %)	29.63	75.93	72.22	46.30	33.33
Relative percent survival (RPS; %)		65.79	60.53	23.68	5.26

## Data Availability

The original contributions presented in the study are included in the article and Appendix; further inquiries can be directed to the corresponding authors.

## Appendix A

**Table A1:** The formulation and proximate composition (g/kg) of the experimental diets used in the present study containing several doses of *Capsicum annuum* oleoresin.

Ingredients	Dietary *Capsicum annuum* Oleoresin Level (g/kg)				
	CT (Control)	C7	C14	C21	C28
Fish meal (Black Sea anchovy) ^a^	250	250	250	250	250
Soybean meal ^a^	270	270	270	270	270
Wheat flour ^a^	177.5	177.5	177.5	177.5	177.5
Wheat gluten ^a^	100	100	100	100	100
Vitamin mixture ^b^	10	10	10	10	10
Mineral mixture ^c^	20	20	20	20	20
BHT ^a^	0.01	0.01	0.01	0.01	0.01
Wheat starch ^a^	49.99	49.99	49.99	49.99	49.99
Fish oil (Black Sea anchovy) ^a^	120.0	113.0	106.0	99.0	92.0
Lysine ^d^	2.5	2.5	2.5	2.5	2.5
*Capsicum annuum* oleoresin ^e^	0.0	7.0	14.0	21.0	28.0
Total (g)	1000	1000	1000	1000	1000
**Chemical analyses (%, on DM basis)**					
Crude protein (CP)	43.01	43.02	43.01	43.00	43.05
Crude lipids (CL)	16.11	16.12	16.10	16.13	16.12
Crude ash	4.75	4.78	4.80	4.82	4.86
Nitrogen-free extract (NFE) ^f^	25.25	25.32	25.36	25.30	25.34
Gross energy (GE; Kj/g) ^g^	20.81	20.82	20.82	20.82	20.84

^a^ These ingredients were purchased from Sibal A.Ş. Sinop (Türkiye). BHT, butylated hydroxytoluene, used as an antioxidant feed supplement. ^b^ Vitamin mixture (composition per kg diet): vitamin A (18,000 IU); vitamin D3 (2500 IU); vitamin E (250 mg); vitamin K3 (12 mg); vitamin B1 (25 mg); vitamin B2 (50 mg); vitamin B3 (270 mg); vitamin B6 (20 mg); vitamin B12 (0.06 mg); vitamin C (200 mg); folic acid (10 mg); calcium d-pantothenate (50 mg); biotin (1 mg); inositol (120 mg) and choline chloride (2000 mg). ^c^ Mineral mixture (composition per kg diet): iron (75.3 mg); copper (12.2 mg); manganese (206 mg); zinc (85 mg); Iodine (3 mg); selenium (0.350 mg) and cobalt (1 mg). ^d^ L-Lysine, L5501, Sigma-Aldrich (Germany). ^e^ *Capsicum annuum* oleoresin was procured from Smart Kimya Tic. ve Danışmanlık Ltd. Şti. (İzmir, Türkiye). ^f^ NFE = dry matter (CL + Crude ash + CP). ^g^ GE was calculated using 23.6 kJ/g protein, 39.5 kJ/g lipid, and 17.0 kJ/g NF.

**Table A2:** Gene-specific primers were employed. Details of the primers used in the qPCR study.

Primers	Primers	Sequence (5′–3′)	NCBI GenBank Accession Numbers	Published in
*il-1* *β*	Forward	GGAGAGGTTAAAGGGTGGCGA	AJ223954.1	[56]
	Reverse	TGCCGACTCCAACTCCAACA		
*IgT*	Forward	AACATCACCTGGCACATCAA	AY870265.1	[57]
	Reverse	TTCAGGTTGCCCTTTGATTC		
*IFN-γ*	Forward	CTGTTCAACGGAAACCCTGT	NM_001160503.1	[57]
	Reverse	AACACCCTCCGATCACTGTC		
*il-8*	Forward	CTCGCAACTGGACTGACAAA	AJ279069.1	[57]
	Reverse	TGGCTGACATTCTGATGCTC		
*SAA*	Forward	GGAGATGATTCAGGGTTCCA	NM_001124436.1	[57]
	Reverse	TTACGTCCCCAGTGGTTAGC		
*TGF-β*	Forward	TCCGCTTCAAAATATCAGGG	X99303.1	[57]
	Reverse	TGATGGCATTTTCATGGCTA		
*β* *-actin*	Forward	ATGGGCCAGAAAGACAGCTACGTG	NM_001124235.1	[58]
	Reverse	CTTCTCCATGTCGTCCCAGTTGGT		
